# An Analysis of the Effects of Smartphone Push Notifications on Task Performance with regard to Smartphone Overuse Using ERP

**DOI:** 10.1155/2016/5718580

**Published:** 2016-06-05

**Authors:** Seul-Kee Kim, So-Yeong Kim, Hang-Bong Kang

**Affiliations:** Department of Media Engineering, Catholic University of Korea, Bucheon-si, Gyeonggi-do 420-743, Republic of Korea

## Abstract

Smartphones are used ubiquitously worldwide and are essential tools in modern society. However, smartphone overuse is an emerging social issue, and limited studies have objectively assessed this matter. The majority of previous studies have included surveys or behavioral observation studies. Since a previous study demonstrated an association between increased push notifications and smartphone overuse, we investigated the effects of push notifications on task performance. We detected changes in brainwaves generated by smartphone push notifications using the N200 and P300 components of event-related potential (ERP) to investigate both concentration and cognitive ability. ERP assessment indicated that, in both risk and nonrisk groups, the lowest N200 amplitude and the longest latency during task performance were found when push notifications were delivered. Compared to the nonrisk group, the risk group demonstrated lower P300 amplitudes and longer latencies. In addition, the risk group featured a higher rate of error in the Go-Nogo task, due to the negative influence of smartphone push notifications on performance in both risk and nonrisk groups. Furthermore, push notifications affected subsequent performance in the risk group.

## 1. Introduction

Smartphones quickly provide users with various types of information wherever they are, at any time, and enable access to various content via social network services (SNS) and mobile games [1]. In addition, alternative technologies have been installed on smartphones to enable access to content such as GPS, music, and photos, which used to be accessible only through specialized devices like navigation devices, MP3 players, and cameras. Smartphones have become established as tools for day-to-day convenience; however, smartphone addiction has emerged as an increasingly prevalent social issue, which in case of overuse interferes with daily life [2–5].

Smartphone addiction refers to a behavioral disorder in which a person uses content such as SNS, Internet browsing, and mobile games for an excessive amount of time without self-control, such that it interferes with daily life in a manner similar to Internet addiction. Overuse of smartphones negatively affects users, causing psychological conditions such as sleep disorder and attention deficit disorder and physical disabilities such as carpal tunnel syndrome and forward head posture [6–8]. In particular, Kim [9] highlighted push notifications from SNS, multimedia message services (MMS), or applications (apps) as important factor to the excessive use of smartphones. Here, a push notification refers to a frequent alarm that provides users with information regardless of whether they want it or not. This is the opposite concept to pull methods, in which the user directly searches for information. With push notifications, the smartphone providing the information also controls the flow of information. While the advantage of push notifications is that information can be instantly delivered, this information is provided regardless of the users need. Kim [9] reported that the duration and frequency of smartphone usage increased with the number of push notifications, concomitant with an increased risk of developing smartphone addiction.

However, since most studies on smartphone addiction or overuse have included questionnaire-based surveys or behavioral observations [6, 10, 11], these results are based on subjective opinions and might therefore be biased by individual perception. It was subsequently difficult to investigate changes in behavioral characteristics or cognitive ability caused by smartphone overuse. Therefore, it is necessary to explore them using objective measurements.

In this paper, we analyze brainwaves to detect the effects of smartphone push notifications on task performance with regard to smartphone overuse. Brainwaves refer to the recording of continuous changes in potential between two points on the scalp, which relate to electrical activity in the brain. Brainwave signals vary according to neural activity, the conditions at the time of measurement, and general brain function. We measured event-related potentials (ERPs) in order to study the brainwaves produced in response to a specific stimulus. In many studies, ERP was used to assess both concentration and cognitive ability using repeated tasks, wherein the same stimulus was presented repeatedly, and differences in the averaged potentials induced by the stimulus were analyzed. Specifically, this study investigated the ERP components N200 and P300, which reflect cognitive functions such as attention and concentration [12, 13]. Next, we measured changes in concentration and cognition ability caused by various levels of smartphone overuse.

## 2. Related Works

### 2.1. Smartphone Addiction

Recent studies have addressed the issue of pathological smartphone use. Existing studies on smartphone addiction mostly consist of surveys based on questionnaires and interviews. However, these studies did not reflect continuous evaluation, and doubts have been raised regarding the objectivity of such evaluations. Darcin et al. [14] defined smartphone addiction in college students with regard to social anxiety and loneliness and reported that people who were younger when they first used smartphone-based SNS exhibited a wider range of addictive smartphone habits. In addition, this study found that psychological tendencies, such as loneliness, were strongly linked to excessive smartphone use.

Moreover, Sayrs [15] reported that cognitive states, including stress, productivity, boredom, and loneliness, were related to smartphone use. Lee et al. [1] monitored the usage of GPS apps and other tools in order to objectively measure patterns of pathological smartphone use, performed statistical analysis of the data, and compared the analysis results with Korean smartphone addiction scale (K-SAS) scores. The results identified a strong correlation between K-SAS results and the data obtained by statistical analysis. Based on these findings, Lee et al. [1] proposed a smartphone addiction management system (SAMS). Accordingly, Lin et al. [16] created a mobile app for the identification of smartphone addiction, which indicated that the frequency of smartphone use was strongly linked to smartphone addiction. Park et al. [17] conducted a survey questionnaire consisting of the following items: motivation for social inclusion, motivation for instrumental use, innovativeness, intention to keep using the smartphone, smartphone dependency, and so forth. As a result, they suggested that smartphone dependency was affected by smartphone usage history.

Furthermore, Lee et al. [18] analyzed smartphone usage patterns and habits in a smartphone risk group and a nonrisk group and reported that the duration and frequency of smartphone use correlated with excessive use. Push notifications for incoming messages were strongly linked to smartphone addiction. Despite little difference in the duration of smartphone use between the risk group and the nonrisk group, they identified that previous studies focused on the duration of use as a cause of addiction rather than a characteristic of use. Therefore, they suggested that characteristics of smartphone use other than duration should be investigated. Similarly, Xu et al. [19] argued that despite the introduction of various mobile devices and apps, the understanding of smartphone usage patterns is lacking compared to that of the existing web services. In addition, Xu et al. [19] investigated the usage patterns of apps at a national level and reported various usage patterns according to a smartphone user's pattern of behavior (time, place, object, etc.).

### 2.2. Auditory Notification

Auditory push notifications are a useful tool for notifying users of incoming data and messages. However, push notifications may also cause stress, depending on the user's environment. Previous studies have evaluated the effects of auditory push notifications on behavior but have failed to consider various factors between users, including subjective and environmental differences. To overcome this limitation, performance evaluation methods that can be applied to various environments have been suggested [20]. Yoon and Lee [21] performed a study into the stress levels of users who received push notifications from a smartphone messenger and proposed a design method for push notifications that might reduce stress. Kim [9] investigated the effects of push notifications on the formation of habits for mobile app use and found that increasing the number of push notifications produced a greater frequency of app visits. These results suggest an effect of push notifications on media habit formation.

### 2.3. ERP

ERPs refer to brainwaves that occur in response to a specific stimulus and reflect brain responses associated with sensory, motor, and cognitive events. ERP-based methods of assessment are widely used in behavioral research, since they provide high temporal resolution of neural processes relating to behavior [22]. Most ERP studies focus on stimulus responses relating to sensory and cognitive abilities. Two important components of ERP analysis are P300 and N200. P300 refers to a peak in the positive direction appearing in the interval between 300 and 350 ms after the presentation of auditory stimuli and between 350 and 450 ms after the presentation of visual stimuli. The maximum amplitude appears at parietal electrode sites, and as a component of ERP, it is an important index for the study of information processing in the brain, with particular relevance to cognitive psychology [23, 24].

P300 is divided into P3a and P3b. P3a usually refers to P300 and exhibits maximum amplitude in the frontal and central regions when nontarget stimuli appear amidst repeated target stimuli. P3a relates to attention processing and features a relatively larger amplitude and shorter latency than P3b. P3b refers to a component that exhibits maximum amplitude in the parietal region in response to target stimuli [25–27]. Since the latency of P300 is typically believed to represent the speed of stimulus classification, a shorter latency reflects superior cognitive ability. The amplitude of P300 reflects stimulus information, wherein a higher level of concentration is accompanied by a larger amplitude. Therefore, a reduction in P300 amplitude is used as an indicator of various psychological symptoms, including alcohol and drug addiction [28].

By contrast, N200 refers to a stimulus-induced negative peak appearing in the interval between 250 and 400 ms (180–325 ms) after stimulus onset. N200 is the greatest in the frontal and central regions and is divided into the following three components: N2a, N2b, and N2c. N2a, also referred to as mismatch negativity (MMN), reflects mismatched responding to a task. MMN is a component induced by an infrequent stimulus that appears amidst a repeated stimulus and is mainly assessed in studies relating to auditory stimuli [13]. Unlike MMN, N2b is not limited to auditory processes but appears in response to both visual and auditory stimuli. N2b is mainly observed using the oddball paradigm and appears in central regions in response to nontarget stimuli. Comparatively, N2c is expressed in posterior regions in response to visual stimuli and in frontal-central regions in response to auditory stimuli. The latency of N2c is an indicator of reaction time in response to the stimulus, and N2c amplitude is larger in response to target stimuli compared to nontarget stimuli. The subcomponents of N200 possess distinct characteristics. N2a does not require concentration on the stimulus and does not appear in conjunction with the P3 component. However, N2b and N2c require concentration on the stimulus and appear together with P3a and P3b, respectively [29].

Cristini et al. [30] used ERPs to assess recurrence risk in patients with alcohol addiction. They reported increased recurrence among patients with alcohol addiction who demonstrated higher P300 amplitudes at the Cz and Pz electrodes. Pandey et al. [31] found that the N200 amplitude of patients with alcohol addiction was not increased between Nogo and Go responses and reported that this was the result of a decline in frontal lobe function. However, no previous study has used ERPs to investigate the differences in response to push notifications with regard to smartphone overuse. Therefore, the aim of this study was to use the Go-Nogo task to study the effects of smartphone push notifications on task performance by analyzing N200 and P300 components [32].

## 3. Research Goals

The aim of this study was to monitor both the amplitude and latency of the ERP components N200 and P300, in order to evaluate the effects of push notifications on task performance, and to identify whether the concentration and cognitive abilities of the smartphone risk group were reduced compared to the nonrisk group. We performed a Go-Nogo task twice, with each round consisting of three sessions. Subjects were given a push notification with a natural intensity during the first session of the first Go-Nogo task. The push notification was also delivered before starting the third session. From this, we investigated the effects of smartphone push notifications before and during the task on subjects' task performance. No push notifications were delivered in the second Go-Nogo task, during which performance was compared depending on the presence or absence of smartphone push notifications.

## 4. Methods

### 4.1. Subjects

Subjects were recruited using an announcement for a study to analyze brainwaves according to the colorfulness of a video, in order to blind them from the nature of the experiment. A presurvey was conducted with questions taken from the smartphone addiction scale (hereafter, S scale) developed by the Korean National Information Society Agency in 2011 [33] and additional questions. Individuals on the boundary between the smartphone risk and nonrisk groups were excluded in order to obtain a distinct difference between the two groups. The experiment took no longer than 2 hours, and subjects were informed about the experiment prior to completion. This excluded the delivery of push notifications but included instructions about filling in a consent form prior to the experiment. Data from 14 subjects was used for analysis, with six in the risk group (3 women and 3 men; mean age, 22 years) and eight in the nonrisk group (5 women and 3 men; mean age, 22.6 years).

### 4.2. Go-Nogo Task

**Figure 1:**
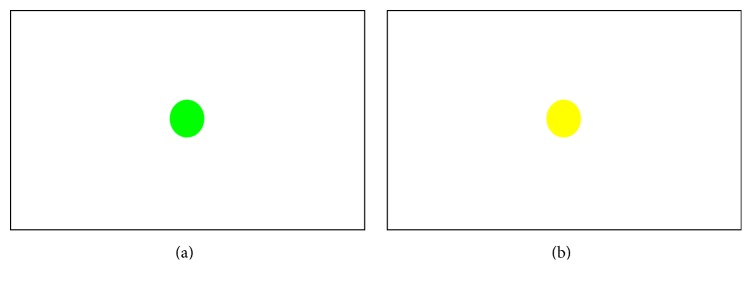
Go-Nogo task: (a) example of Go task and (b) example of Nogo task.

Various figures were presented in the center of a screen with a white background, as shown in Figure 1, and trials were designed so that subjects pressed number 1 when the color of the figure was yellow and pressed nothing when it was green. The length of stimulus presentation was set to 150 ms, and each session was composed of 186 trials, with 148 Go trials and 38 Nogo trials. Subjects were allowed 1 min of practice prior to the main trial, which was composed of three sessions. Each session lasted for approximately 6 min, with 1 min rest between sessions. Therefore, task performance lasted for a total of approximately 18 min.

### 4.3. Push Notification

**Figure 2:**
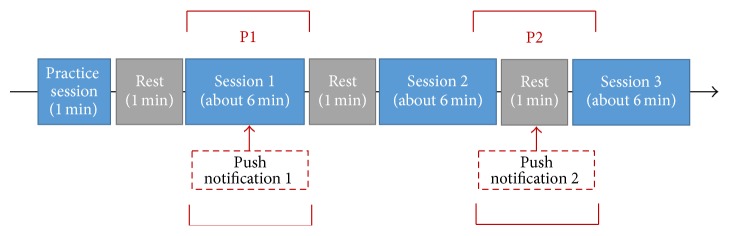
Go-Nogo task procedure.

This experiment used vibration push notifications for all subjects in order to prevent differences in response relating to the smartphone notification sound. Push notifications were delivered twice during the experiment. This is shown in Figure 2. The first push notification (hereafter, P1) was presented during performance of the Go-Nogo task in Session 1, and the second push notification (hereafter, P2) was presented during rest time before starting the Go-Nogo task in Session 3. Push notifications were delivered via a special device, placed behind the subjects so that they were clearly able to recognize the push notification but were not able to check it.

### 4.4. EEG (Electroencephalogram) Procedure

**Figure 3:**
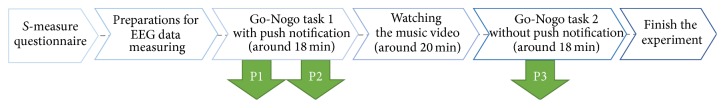
Experimental procedure.

Subjects wore an EEG cap during the experiment to record brainwaves. The experiment was composed of three steps: Task 1 with the push notification, watching a video, and Task 2 without the push notification (hereafter, P3). This is shown in Figure 3. Approximately 18-minute-long Go-Nogo tasks were performed during Tasks 1 and 2. Two push notifications (P1 and P2) were delivered when performing Task 1. Subjects watched a video during the rest between the first and the second tasks. A null push notification (P3) was delivered during the second task so that the data could be used as a control. In all experiments, the subjects performed the tasks in a separate space to the tester with illumination intensity of 0, so that they could focus on the task.

## 5. Data Analysis

### 5.1. ERP (Event-Related Potential)

**Figure 4:**
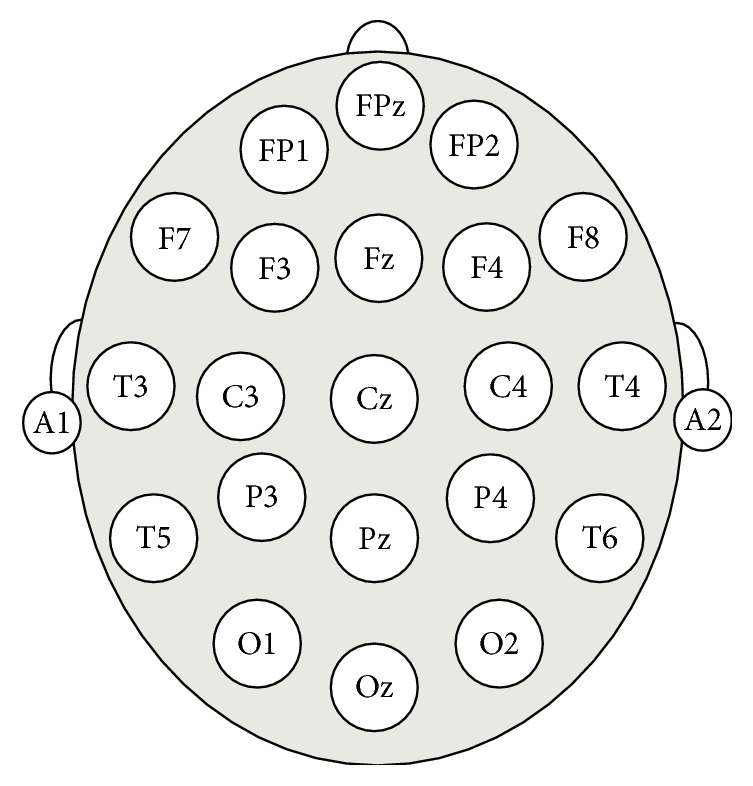
International 10-20 electrode placement system.

For ERP analysis, N200 and P300 were analyzed for Nogo responses in order to examine the differences in task performance between the smartphone risk group and the nonrisk group following the administration of push notifications. A NeuroScan device and the Curry 7 software program were used for the measurement of brainwaves. To minimize noise in the experiment, brainwave data were recorded after impedance was set to 5 or below, while electrodes with an impedance of 10 or over at the end of the experiment were excluded from analysis. Brainwaves were measured based on 10-20 system as shown in Figure 4. Of the 64 channels, FCz in the central frontal region was selected as the representative electrode for ERP measurement, since N200 and P300 could be easily measured and there was less noise in the data. To remove noise from the ERP data, the pre-200 ms and post-800 ms domains in the area outside of the threshold (min 0, max 60) were substituted for the covariance values and the baseline was set to constant. A mean ERP plot of the interval between −200 ms and 500 ms was derived from 38 Nogo trials in each session. Since it was difficult to verify the normal distribution of the amplitude and latency data, we conducted nonparametric statistical testing using the Mann-Whitney *U* test.

### 5.2. Go-Nogo Task

The two tasks were compared with regard to differences in performance based on smartphone push notifications. First, in order to identify differences according to the timing of push notifications, performance in P1, during which the push notification was delivered during the task, was compared with that of P2, where the push notification was delivered prior to the task. Second, in order to ascertain differences according to the presence or absence of push notifications, P1 performance with push notifications and P3 performance without push notifications were compared. Here, performance referred to error rate and reaction time. The error rate was defined as the rate of Go responses in Nogo trials and the number of Nogo responses within Go trials across all sessions, wherein reaction time referred to the response time in Go trials.

## 6. Results

### 6.1. ERP (Event-Related Potential)

**Figure 5:**
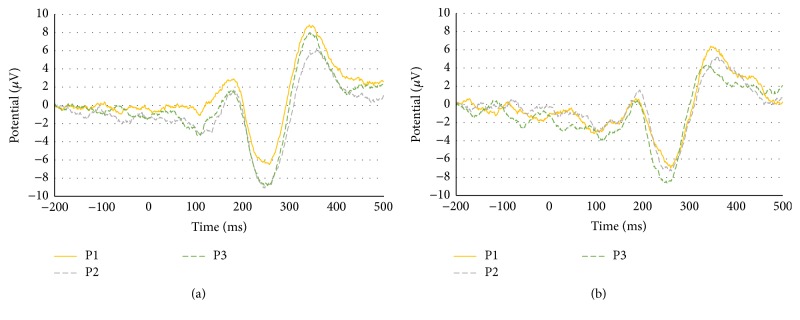
Results of the between-sessions, within-group comparisons: (a) nonrisk group and (b) risk group.

**Figure 6:**
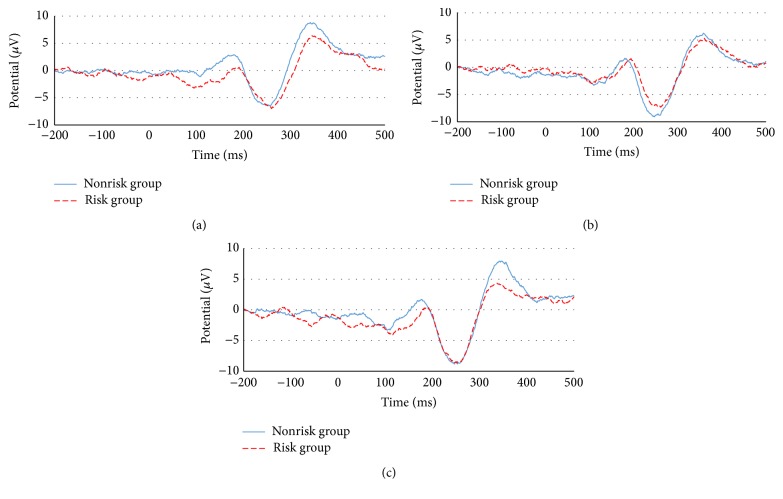
Comparison between the nonrisk group and the risk group in each session: (a) P1 session, (b) P2 sessions, and (c) P3 sessions.

ERP data were compared between sessions within each group and between the risk and the nonrisk groups for each session. The results of the between-session, within-group comparisons are presented in Figure 5, where the yellow, gray, and green lines show the ERPs for P1, P2, and P3, respectively. Results for the comparison between the risk and nonrisk groups in each session are presented in Figure 6, where the blue and red lines indicate the nonrisk and the risk group, respectively. The N200 amplitude and latency values are presented in Table 1.

When the amplitudes of the risk group were compared by session, the lowest amplitude (−6.99 *μ*V) was detected in P1, in which the push notification was presented during the task, and the second lowest amplitude (−7.315 *μ*V) was detected in P2, in which the push notification was presented before starting the task. P1 (261 ms) and P2 (258 ms) featured similarly long latencies, whereas P3 had a shorter latency (253 ms). These data indicate that P1, in which push notifications were presented during the task, had the greatest impact on concentration and cognitive ability in the risk group.

In the nonrisk group, similar to the risk group, P1 featured the lowest amplitude (−6.503 *μ*V), whereas the amplitudes of P3 (−8.834 *μ*V) and P2 (−9.086 *μ*V) were similar. Latency values were 258 ms for P1, 248 ms for P2, and 247 ms for P3, wherein P1 demonstrated the longest latency, indicating that concentration and cognitive ability declined in the nonrisk group following push notification delivery during the task, whereas notifications before the task had no effect on ability. When the nonrisk group and the risk group were compared in each session, the amplitude of P1 (−6.503 *μ*V) in the nonrisk group was smaller than that of the risk group (−6.99 *μ*V). For P2 and P3, by contrast, the amplitudes of the risk group (−7.315 *μ*V, −8.635 *μ*V) were lower than those of the nonrisk group (−9.086 *μ*V, −8.834 *μ*V). In addition, the risk group demonstrated longer latencies than the nonrisk group in all sessions. This indicates that push notifications delivered during the task affected the nonrisk group; however, subsequent task performance was not affected.

By contrast, the risk group was continuously affected by push notifications delivered during the task, even during subsequent task performance. The amplitude and latency of P300 are presented in Table 2. P300, unlike N200, exhibited no particular trends with regard to amplitude or latency in either session for the risk group or the nonrisk group. When the risk and nonrisk group were compared across sessions, P1, P2, and P3 in the risk group exhibited lower amplitudes than those in the nonrisk group, and P1 and P2 featured longer latencies. These data indicate that the risk group was more affected by push notifications than the nonrisk group.

### 6.2. Go-Nogo Task

**Figure 7:**
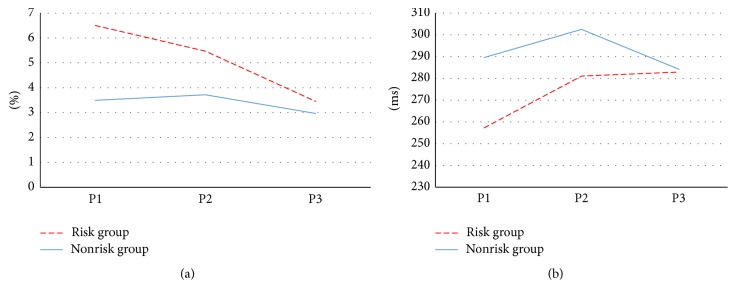
Results of Go-Nogo task: (a) error rate and (b) reaction time.

Error rate and reaction time for the Go-Nogo task are shown in Figure 7. The risk group featured higher error rate values overall. Performance during P1, when push notifications were delivered during the task, featured the highest error rate. The nonrisk group showed similar error rates for P1, P2, and P3. The risk group exhibited shorter reaction times than the nonrisk group. In addition, no significant differences were identified in reaction time or error rate between P1, P2, and P3 in the nonrisk group, whereas the risk group exhibited shorter reaction times and higher error rates in P1, which then stabilized in P2 and P3. This indicates that the risk group made hastier decisions during P1 than the other sessions, due to the effects of push notification delivery on task performance.

## 7. Discussion

Subjects in the present study performed tasks composed of repeated trials to investigate the effects of push notifications between a smartphone risk group and a nonrisk group. The two tasks were divided into three subsessions (P1, P2, and P3) with varying conditions. Previous studies [3–5] indicated the effects of smartphone addiction using surveys or psychological and behavioral observations. It was possible to determine the effects of smartphone addiction or overuse on physical or psychological health by the users' responses. However, such studies might generate superficial results due to the subjective nature of previous research. Therefore, it is necessary to investigate the physiological effects of smartphone overuse in terms of changes in brainwaves.

To address this problem, we analyzed changes in brainwaves relative to physiological effects or cognitive ability. From the ERP experimental results, we found that differences in concentration and cognition ability correlated with levels of smartphone overuse. Lee et al. [18] found a correlation between smartphone addiction and push notifications. In our study, the smartphone overuse group (the risk group) was more sensitive to push notifications than the nonrisk group. In particular, the risk group demonstrated impaired concentration after hearing the push notification, an observation that was not detected in the nonrisk group.

However, our experiments had several limitations. First, since only FCz electrodes were used in the present study, we were unable to investigate the reaction of parietal or occipital regions. If we compared brainwaves at additional electrode positions, it would be possible to investigate the functional influence of smartphone overuse in various brain regions. In addition, this study utilized the same Go-Nogo task in all sessions to create an experimental environment in which only the push notifications were not controlled.

## 8. Conclusion

In this paper, we explored the effects of smartphone push notification delivery during a task according to the level of smartphone overuse using ERP. From our experimental results, we found that both the smartphone risk group and the nonrisk group demonstrated sensitive reactions to smartphone push notifications during tasks. While the performance of the nonrisk group was unaffected by previously delivered push notifications, the delivery of push notifications affected subsequent task performance in the risk group. In other words, smartphone push notifications produced a decline in task performance in the smartphone risk group, exerting a negative influence on cognitive function and concentration.

ERP was able to measure the negative effects of smartphone overuse in terms of psychological or physical characteristics. Particularly in the risk group, we observed lower N200 amplitude and longer response latency. A higher error rate and longer reaction time were also identified in the risk group during the Go-Nogo task.

In future studies, it might be possible to determine the level of smartphone overuse by measuring responses to push notifications. While this study used a single type of push notification, future studies using various types will be required to investigate sensitivity to different push notifications.

## Figures and Tables

**Table 1:** N200 amplitude and latency.

	Nonrisk group			Risk group		

Session	P1	P2	P3	P1	P2	P3

Amplitude (*μ*V)	−6.50376	−9.08664	−8.83492	−6.99048	−7.3151	−8.63528
Latency	258	248	247	261	258	253

**Table 2:** P300 amplitude and latency.

	Nonrisk group			Risk group		

Session	P1	P2	P3	P1	P2	P3

Amplitude (*μ*V)	8.83268	6.27596	7.96026	6.410275	5.35475	4.364368
Latency	343	359	347	347	361	338
